# Laparoscopic bowel resection of deep infiltrating endometriosis. Comparative outcomes of a public teaching hospital and a referral private hospital^1^

**DOI:** 10.1590/s0102-865020200090000008

**Published:** 2020-09-23

**Authors:** Rogério Serafim Parra, Marley Ribeiro Feitosa, Fernando Passador Valerio, Hugo Parra de Camargo, José Vitor Cabral Zanardi, Omar Feres, José Joaquim Ribeiro da Rocha, Júlio César Rosa-e-Silva

**Affiliations:** I PhD, Division of Coloproctology, Department of Anatomy and Surgery, Faculdade de Medicina de Ribeirão Preto, Universidade de São Paulo (FMRP-USP), Brazil. Conception and design of study; acquisition, analysis and interpretation of data; manuscript writing; critical revision; final approval.; II PhD, Department of Anatomy and Surgery, FMRP-USP, Ribeirao Preto-SP, Brazil. Analysis and interpretation of data, statistics analysis, final approval.; III MD, Department of Gynecology and Obstetrics, FMRP-USP, Ribeirao Preto-SP, Brazil. Acquisition, analysis and interpretation of data; final approval.; IV PhD, Department of Anatomy and Surgery, FMRP-USP, Ribeirao Preto-SP, Brazil. Acquisition, analysis and interpretation of data; final approval.; V PhD, Department of Gynecology and Obstetrics, FMRP-USP, Ribeirao Preto-SP, Brazil. Analysis and interpretation of data, final approval.; VI PhD, Associate Professor, Division of Coloproctology, Department of Anatomy and Surgery, FMRP-USP, Ribeirao Preto-SP, Brazil. Analysis and interpretation of data, critical revision, final approval.; VII PhD, Associate Professor, Head, Division of Coloproctology, Department of Anatomy and Surgery, FMRP-USP, Ribeirao Preto-SP, Brazil. Conception and design of the study; acquisition, analysis and interpretation of data; critical revision; final approval.; VIII PhD, Associate Professor, Department of Gynecology and Obstetrics, FMRP-USP, Ribeirao Preto-SP, Brazil. Analysis and interpretation of data, critical revision, final approval.

**Keywords:** Laparoscopy, Endometriosis, Public Health, General Surgery, Outcome Assessment, Health Care

## Abstract

**Purpose:**

To compare the operative outcomes of laparoscopic surgical treatment for bowel endometriosis in a public teaching hospital versus in a private referral hospital.

**Methods:**

The indications for surgery, type and time of operation, length of hospital stay, need for a temporary stoma, rate of conversion to open surgery, and postoperative complications were evaluated.

**Results:**

One hundred eighty-one patients were included (150 patients, 82.9%, in a private hospital). In the private hospital, there were more patients with infertility [56% vs. 29%; P=0.01] as an indication for surgery) and segmental resection was more common in the private hospital (48% vs. 29%, p=0.05). The average operative time (211.9±83.4 minutes vs. 128 ± 55 minutes, p<0.001) as well as the length of hospital stay (3.97±1.7 days vs. 1.56±0.85 days, p<0.001) was higher in the public hospital; the rate of conversion to open surgery was significantly lower in the private hospital (2% vs. 32.3%, p<0.001). Operations performed at the public hospital were associated with higher rates of postoperative complications (Clavien-Dindo II and II) (38.7% x 11.3%, p=0.021; OR 3.2, CI 95% 1.2-8.0).

**Conclusion:**

Laparoscopic surgery in private centers was associated with reductions in major complications, surgical times, lengths of stay and rates of conversion to open surgery compared to that in public teaching hospitals.

## Introduction

Endometriosis is a chronic inflammatory disease with a high prevalence among women of reproductive age, especially infertile women, and among women presenting with chronic pelvic pain^1^. Deep infiltrating endometriosis (DIE) is defined as infiltrating lesions greater than 5 mm in depth and is one of the most severe types of endometriosis. DIE frequently presents with nodules involving the rectovaginal space, bladder, pelvic nerves, ureters, and the bowel, particularly the rectosigmoid. Lesions can be single or multifocal, and depending on the anatomical site affected, they can involve symptoms that vary from dysmenorrhea to dyspareunia, dyschezia, and rectal bleeding^2,3^.

Some factors should be considered for the management of DIE with bowel involvement, such as patient age, pain intensity, risk of intestinal obstruction and desire for pregnancy^4,5^. Surgery is mainly indicated in patients with pelvic pain who do not respond to medical therapy and in patients who want to become pregnant^6,7^. There are several surgical techniques, such as laparoscopic segmental colon resection, rectal shaving, and rectal disc excision, to treat DIE infiltrating the bowel. The complete excision of all endometriotic lesions is the main objective of laparoscopic surgery, which requires a multidisciplinary approach^6,7^.

Disparities in healthcare services for women with endometriosis with public versus private health insurance have been previously described. Studies have reported significant differences in the use trends of endometriosis-related medical services and prescriptions, indicating differences in healthcare access based on socioeconomic parameters^8^. In our country, there are reports of difficulties accessing image exams, such as transvaginal ultrasound for deep endometriosis and magnetic resonance (MRI), in addition to difficulties accessing laparoscopy in public hospitals^9^. In addition, laparoscopic surgical procedures in public teaching hospitals are performed by resident physicians at the beginning of their careers when they have little or no experience.

There are some comparisons between public and private hospitals; in one study, the authors analyzed the frequency of surgical and medical complications after resection of colorectal cancer (CRC) between a public tertiary referral hospital and a private hospital^10^. In the study, the type of hospital had no impact on the rates of specific complications apart from septicemia and cardiorespiratory complications, which were higher in the public hospital. However, there are no precise data comparing public and private health services regarding the length of stay, surgical time, conversion rates to open surgery, postoperative complications, and a need for a temporary stoma for surgery for DIE with bowel involvement. The aim of this study was to compare the operative and postoperative outcomes of laparoscopic surgical treatment for bowel endometriosis in two centers: a public teaching hospital and a private referral hospital.

## Methods

### *Study design and data collection*

This study was approved by the ethical Institutional Review Board from Hospital São Paulo, Ribeirao Preto (CS.02/2019, 10.Oct.2019), and by the ethical Institutional Review Board from HCFMRP-USP (CAAE: 31679420.3.0000.5440, Ethics Committee number 4.029.839/2020). The study protocol conforms to the ethical guidelines of the 1975 Declaration of Helsinki as reflected in a priori approval by the institution’s human research committee.

We conducted a retrospective analysis between October 2014 and October 2019, using the database of the Clinics Hospital, Faculdade de Medicina de Ribeirão Preto, Universidade de São Paulo (public teaching hospital) of patients with DIE who underwent laparoscopy with bowel resection (laparoscopic shaving for bowel endometriosis, rectal disc excision, and segmental ileal and/or colon resection). We chose this period of 5 years for the retrospective analysis in the public hospital, because there are data prospectively recorded in the database of Hospital São Paulo, Ribeirao Preto-SP (private referral hospital) in the same period. We evaluated the following patient characteristics and compared with two hospitals: age, body mass index (BMI), surgical indication, operation type, operative time, hospital stay length, temporary stoma requirement, conversion rate to open surgery, and postoperative complications, according to the Clavien-Dindo classification^11^. Patients who underwent open surgery as the main surgery were excluded.

### *Statistical analysis*

Categorical variables were expressed as frequencies/percentages, and continuous variables were expressed as the means ± standard deviations. The one-sample Kolmogorov-Smirnov test was used to assess the normality of continuous variables. ANOVA was used to compare continuous variables. Fisher’s exact test or the χ2 test was used to compare categorical variables. A univariate analysis was conducted to identify factors associated with moderate/severe postoperative complications. A multivariate analysis was not conducted since only one predictor of complication was observed. All p values were 2-sided, and a significance level of 5% was established. Statistical analysis was performed with IBM® SPSS® Statistics 20 (IBM SPSS, Costa Mesa, CA).

## Results

### *Clinical demographic data*

A total of 181 women (n=150, 82.9%, private hospital; n=31, 17.1%, public hospital) who underwent laparoscopic surgical management for DIE with bowel involvement were included. The mean age was similar between the patients at the two hospitals (37.3±5.3 years, private *vs.* 37.0±4.7 years, public, p=0.99), as was the rate of patients who had previously received surgical management for endometriosis (61.3% *vs.* 51.6%, p=0.32). Compared to patients at the public hospital, the mean body mass index was lower in patients at the private hospital (25.1±3.8 kg/m^2^ *vs.* 28.3±6.0 kg/m^2^, p<0.001), and more patients were nullipara (74% *vs.* 41.9%, p=0.001).

In the public hospital, the surgical indications were dysmenorrhea (100%) and severe chronic pelvic pain refractory to medical management (96.8%), followed by dyspareunia (77.4%). In the private hospital, the most frequent surgical indication was severe chronic pelvic pain refractory to medical management (66%), followed by infertility (56%), dyspareunia (46%), and dysmenorrhea (34.7%). In the comparison between the two groups, patients undergoing surgery in the public hospital had significantly more cases of pelvic pain (96.8% *vs*. 66%, p<0.001), dysmenorrhea (100% *vs*. 34%, p<0.001), and dyspareunia (77.4% *vs*. 46%, p=0.002) as the reasons for surgery. In the private hospital, there were higher rates of infertility (56% *vs*. 29%, p=0.01) as an indication for surgery. The baseline clinical demographic data are shown in Table 1.

**Table 1:** Baseline clinical characteristics.

Characteristic	All patients N=181	Private N=150 (82.9%)	Public N=31 (17.1%)	P*
**Age**				
Mean±SD (years)	35.6±5.2	35.1±5.3	37.0±4.7	0.28
**BMI**				
Mean±SD (kg/m^2^)	25.7±4.4	25.1±3.8	28.3±6.0	<0.001
**Indication**				
Chronic pelvic pain	129 (71.5%)	99 (66.0%)	30 (96.8%)	<0.001
Adenomyosis	36 (19.9%)	27 (18%)	10 (32.3%)	0.08
Dysmenorrhea	82 (45.3%)	52 (34.7%)	31 (100%)	<0.001
Infertility	93 (51.4%)	84 (56%)	9 (29.0%)	0.01
Dyspareunia	93 (51.4%)	69 (46.0%)	24 (77.4%)	0.002
**Nulliparity**	124 (68.5%)	111 (74%)	13 (41.9%)	0.001
**Previous surgery**				
(for endometriosis)	108 (59.7%)	92 (61.3%)	16 (51.6%)	0.32

SD, standard deviation. BMI, body mass index. *p-value calculated by Fisher exact test or ANOVA.

### *Types of operations*

The rate of segmental resection, which is resection of the rectosigmoid, was higher at the private hospital than at the public hospital (48% *vs*. 29%, p=0.05). In 4.2% (n=3) of patients at the private hospital, the surgical specimen was removed through the vagina (natural orifice specimen extraction, or NOSE), and in the public hospital, none of the patients underwent NOSE. Rectal disc excision (38.7% *vs*. 30%, p=0.39) and shaving for bowel endometriosis (32.3% *vs*. 24%, p=0.16) were not significantly different between the two groups (public vs private). These results are shown in Table 2. The associated surgical procedures performed in private hospitals were hysterectomy (n=19, 12.7%), appendectomy (n=16, 10.7%), ureter nodule excision (n=8, 5.3%) and ileocolic and/or small bowel resection (n=9, 6%). In the public hospital, there were hysterectomies (n=4, 12.9%), appendectomies (n=1, 3.2%), and ureter nodule excisions (n=3, 9.7%). Concomitant surgical procedures are described in Table 3.

**Table 2:** Types of operations.

Operation	All patients N=181	Private N=150 (82.9%)	Public N=31 (17.1%)	P*
Shaving	41 (22.7%)	36 (24.0%)	10 (32.3%)	0.16
Discoid excision	56 (30.9%)	45 (30.0%)	12 (38.7%)	0.39
Segmental resection	82 (45.3%)	72 (48.0%)	9 (29.0%)	0.05

*p-value calculated by Fisher exact test.

**Table 3:** Concomitant surgical procedures.

	Private	Public
Operation, n (%)	150 (82.9)	31 (17.1)
**Shaving**	**36 (24.0)**	**10 (32.3)**
Shaving + Segmental resection	1 (0.67)	1 (3.2)
Shaving + Rectal disc excision	3 (2.0)	1 (3.2)
Shaving + Appendectomy	4 (2.7)	1 (3.2)
Shaving + Histerectomy	5 (3.3)	1 (3.2)
Shaving + Ureter nodule excision	2 (1.3)	1 (3.2)*
**Rectal disc excision**	**45 (30.0)**	**12 (38.7%)**
Rectal disc excision + Segmental resection	1 (0.67)	0 (0)
Rectal disc excision + Appendectomy	5 (3.3)	0 (0)
Rectal disc excision + Histerectomy	6 (4.0)	2 (6.4)
Rectal disc excision + Ureter nodule excision	1 (0.67)	0 (0)
Rectal disc excision + Ileal resection	1 (0.67)	0 (0)
**Segmental resection (rectosigmoid)**	**72 (48.0)**	**9 (29.0%)**
Segmental resection + Appendectomy	7 (4.7)	0 (0)
Segmental resection + Histerectomy	8 (5.3)	1 (3.2)
Segmental resection + Ureter nodule excision	5 (3.3)	3 (9.7)*
Segmental resection + Ileal resection	3 (2.0)	0 (0)
Segmental resection + Ileocecal resection	3 (2.0)	0 (0)
***Ileal resection (without rectal involvement)***	***2 (1.3)***	***0 (0)***

*Three patients (9.7%) in public hospital underwent ureter nodule excision. However, one of the patients underwent shaving + segmental resection + ureter nodule excision.

### *Surgical outcomes*

The main surgical outcomes are described in Table 4. The average operative time (128 ± 55 minutes *vs.* 211.9±83.4 minutes, p<0.001), as well as the length of hospital stay (1.56±0.85 days *vs.* 3.97±1.7 days, p<0.001), was lower for private hospitals. The rate of conversion to open surgery (2% *vs.* 32.3%, p<0.001) was significantly lower at the private hospital. The average follow-up time (28.9±16.7 months *vs.* 21.4±19.9, p=0.01) was higher at the private hospital than the public hospital.

**Table 4:** Main surgical outcomes and follow-up.

Outcome	All patients (N=181)	Private (N=150)	Public (N=31)	P*
**Surgery duration**				
Mean±SD (minutes)	142.1±67.9	128.0±55.0	211.9±83.4	<0.001
**Hospitalization**				
Mean±SD (days)	1.97±1.38	1.56±0.85	3.97±1.7	<0.001
**Postoperative complication**				
Grade II	15 (8.3%)	10 (6.7%)	7 (22.6%)	0.001
Grade III	11 (6.1%)	7 (4.7%)	5 (16.1%)	0.005
**Conversion to laparotomy**	12 (6.6%)	2 (1.3%)	10 (32.3%)	<0.001
Shaving	3 (1.6%)	0	3 (9.7%)	
Disc excision	6 (3.1%)	1 (0.6%)	4 (12.9%)	
Segmental resection	4 (2.2%)	1 (0.6%)	3 (9.7%)	
**30-day readmission rate**	7 (3.9%)	5 (3.3%)	1 (3.2%)	1.000
Shaving	2 (1.1%)	1 (0.6%)	1 (3.2%)	
Disc excision	0	0	0	
Segmental resection	4 (2.2%)	4 (2.7%)	0	
**30-day reoperation rate**	5 (2.8%)	3 (2.0%)	3 (9.7%)	0.09
Shaving	3 (1.6%)	1 (0.6%)	2 (6.4%)	
Disc excision	2 (1.1%)	1 (0.6%)	1 (3.2%)	
Segmental resection	1 (0.6%)	1 (0.6%)	0	
**Need of temporary stoma**	5 (2.8%)	4 (3.3%)	2 (6.4%)	0.27
Shaving	1 (0.6%)	1 (0.6%)	0	
Disc excision	4 (2.2%)	2 (1.3%)	2 (6.4%)	
Segmental resection	2 (1.1%)	1 (0.6%)	0	
**Follow-up**				
Mean±SD (months)	28.6±17.3	30.1±16.4	21.4±19.9	0.01

SD, standard deviation. BMI, body mass index. *p-value calculated by Fisher exact test or ANOVA. Grade II or III according to Clavien-Dindo classification.

Grade II complications were lower in patients at the private hospital (n=10, 6.7% *vs.* n=7, 22.6%; P=0.001). At the private hospital, one patient who underwent disc excision had excessive rectal bleeding within 24 hours of the postoperative period and required blood transfusion; five patients had surgical site infections; one patient had deep vein thrombosis and was successfully treated clinically; two patients had diarrhea within 30 days postoperatively and required hospitalization and intravenous antibiotics; and one patient developed bladder atony. At the public hospital, there were three cases of hemorrhage requiring blood transfusion; one patient had surgical site infections; one patient developed anastomosis stenosis requiring endoscopic dilatation; one patient had postoperative paralytic ileus and prolonged hospital stay; and one patient developed bladder atony. These results are shown in Table 5.

**Table 5:** Grade II complications, according to Clavien-Dindo classification.

Grade II complications*	Private (n=10, 6.7%)	Public (n=7, 22.6%)
**Hemorrhage requiring transfusion, n (%)**	**1 (0.6%)**	**3 (9.7%)**
Shaving	0	0 (1.0%)
Disc excision	1 (0.6%)	2 (6.4%)
Segmental resection	0	1 (3.2%)
**Surgical site infections, n (%)**	**5 (3.3%)**	**1 (3.2%)**
Shaving	0	0
Disc excision	0 (0)	0
Segmental resection	5 (3.3%)	1 (3.2%)
**Deep vein thrombosis, n (%)**	**1 (0.6%)**	**0 (0%)**
Shaving	0	0
Disc excision	1 (0.6%)	0
Segmental resection	0	0
**Diarrhea requiring hospitalization, n (%)**	**2 (1.3%)**	**0 (0%)**
Shaving	0	0
Disc excision	0	0
Segmental resection	2 (1.3%)	0
**Bladder atony, n (%)**	**1 (0.6%)**	**1 (3.2%)**
Shaving	0	0
Disc excision	1 (0.6%)	0
Segmental resection	0	1 (3.2%)
**Anastomosis stenosis, n (%)**	**0 (0%)**	**1 (3.2%)**
Shaving	0	0
Disc excision	0	0
Segmental resection	0	1 (3.2%)
**Paralytic ileus, n (%)**	**0 (0%)**	**1 (3.2%)**
Shaving	0	0
Disc excision	0	1 (3.2%)
Segmental resection	0	0

Grade III complications requiring surgical intervention (n=7, 4.7% *vs.* n=5, 16.1%; P=0.005) were less frequent in patients at the private hospital than at the public hospital. In the private hospital group, two patients had thermal injuries of the rectum during laparoscopy, and both cases were detected within 36 hours of the first surgery; one patient developed a pelvic abscess, with the need for another laparoscopy 47 days after the first surgery; in three patients, there was an accidental injury to the ureter detected during laparoscopy; and one patient presented with a urinoma, and a late thermal bladder injury was detected on the 17th postoperative day requiring a laparoscopic surgical re-approach and ureter reimplantation. In the public hospital, one patient had dehiscence of anastomosis after disc excision detected two days after surgery, requiring laparotomy and stoma; one patient had accidental injury to the ureter detected during laparoscopy; one patient presented with a urinoma, and a late thermal bladder injury was detected on the 14th postoperative day requiring a laparotomy surgical re-approach and ureter reimplantation; one patient had massive postoperative hemorrhage requiring laparotomy; and one patient had an accidental injury of the left iliac vein requiring laparotomy and vascular intervention. The major postoperative surgical complications are presented in Table 6.

**Table 6:** Major complications, classified as Grade III of The Clavien-Dindo classification.

Grade III (major) complications*	Private (n=7, 4.7%)	Public (n=5, 16.1%)
**Thermal bowel injury, n (%)**	**2 (1.3%)**	**0 (0%)**
Shaving	1 (0.6%)	0
Disc excision	1 (0.6%)	0
Segmental resection	0	0
**Pelvic abscess, n (%)**	**1 (0.6%)**	**0 (0%)**
Shaving	0	0
Disc excision	0	0
Segmental resection	1 (0.6%)	0
**Urinary complications, n (%)**	**4 (2.7%)**	**2 (6.4%)**
Shaving	0	1 (3.2%)
Disc excision	0	1 (3.2%)
Segmental resection	4 (2.7%)	0
**Massive hemorrhage, n (%)**	**0**	**1 (3.2%)**
Shaving	0	1 (3.2%)
Disc excision	0	0
Segmental resection	0	0
**Iatrogenic iliac vein injury, n (%)**	**0 (0%)**	**1 (3.2%)**
Shaving	0	0
Disc excision	0	1 (3.2%)
Segmental resection	0	0
**Dehiscence of anastomosis**	**0 (0%)**	**1 (3.2%)**
Shaving	0	0
Disc excision	0	1 (3.2%)
Segmental resection	0	0

We observed that operations performed at the public hospital were associated with higher rates of postoperative complications (Clavien-Dindo II and II) (38.7% x 11.3%, p=0.021; OR 3.2, CI 95% 1.2-8.0) (Table 7).

**Table 7:** Univariate analysis of factors associated with postoperative complications.

Variable	Category	Complication	P	OR	CI (95%)
Age	<35years	10 (12.3%)	0.529	1.3	0.5-3.1
	≥35years	16 (16.0%)			
BMI	<25 kg/m^2^	10 (11.6%)	0.397	1.5	0.6-3.6
	≥25 kg/m^2^	16 (16.8%)			
Symptom	Chronic Pain	21 (16.3%)	0.349	1.8	0.6-5.1
	Other	5 (9.6%)			
Previous surgery	Yes	14 (13.0%)	0.524	0.7	0.3-1.7
	No	12 (16.4%)			
Surgery type	Conservative*	12 (12.1%)	0,398	1.4	0.6-3.4
	Segmental	14 (17.1%)			
Hospital	Public	12 (38.7%)	0.021	3.2	1.2-8.0
	Private	17 (11.3%)			

BMI, body mass index. OR, odds ratio. CI, confidence interval. *Conservative: rectal shaving and/or disc excision.

The 30-day reoperation rates (2.0% *vs.* 9.7%, p=0.01) were lower for private hospitals than for public hospitals. The 30-day readmission and the need for a temporary stoma were not significantly different between the two groups. The private hospital had a higher average follow-up time (28.9±16.7 months *vs.* 21.4±19.9 months; P=0.01) than that of the public hospital.

## Discussion

Our study showed that laparoscopic surgery for the treatment of DIE with bowel involvement in a private referral hospital compared to that in a public hospital was associated with decreased operation times, lower conversion rates to open surgery and lower rates of postoperative complications, especially major complications. Chronic pelvic pain, dysmenorrhea and dyspareunia were more frequent in patients at the public hospital, while infertility was more common as an indication for surgery at the private hospital. The mean follow-up was higher for the private hospital than the public hospital.

Although some clinical characteristics were similar between the two groups, such as average age and rate of previous surgery, other characteristics were different, such as BMI and main symptoms before surgery. There are some possible reasons why patients in public hospitals have higher rates of pain as the main indication for surgery. There is a delay in the diagnosis of endometriosis, and the interval between the onset of symptoms and diagnosis is longer, which makes the referred cases more symptomatic^9,12,13^. The delay in the diagnosis of endometriosis is too long, especially for young women with pelvic pain^9^. In addition, there is also a delay in the referral of patients with DIE in the public system, and due to this, the cases that arrive at teaching hospitals might be more advanced. There is a lack of awareness and lack of knowledge of physicians about endometriosis, and more information relating to endometriosis should be offered to general physicians and gynecologists to reduce the time taken to diagnose this condition^14^. Decreasing the diagnostic delay requires increased patient education, timely referral to a women’s healthcare provider and a change in physician approach. The time has come to reduce disparities and to minimize delays in the diagnosis and treatment of endometriosis for the benefit of women worldwide.

Another reason for the differences between hospitals might be due to the unavailability of easy access to specific exams, such as transvaginal ultrasound for DIE and/or MRI of the pelvis; only very highly symptomatic patients undergo surgery in the public system. Finally, in public tertiary hospitals, there is a preferential scheduling of oncological surgeries, and the queue for scheduling endometriosis surgeries (which is considered a benign disease) is very long. Thus, the indication for surgery ends up being reserved for more advanced or more symptomatic cases.

We observed that in the private hospital, the rate of infertile patients was higher than in the public hospital. Despite many of our private patients being referred, in a private setting, there is easier access to medical care. In addition, medical treatments that act through hormonal modulations (hormonal contraceptives, progestogens, anti-progestogens, GnRH analogs and antagonists, and aromatase inhibitors) can block ovulation and culminate in a hypoestrogenic microenvironment^15^. Therefore, this option is inappropriate for patients who have infertility associated with endometriosis and who wish to conceive normally^16^. Finally, there is an earlier endometriosis diagnosis in many cases at private hospitals^17^. In the United States, for instance, the time to endometriosis diagnosis appears to have shortened, mainly due to better patient and physician education regarding symptomatology. It is different for the public health system in Brazil. On the other hand, at the public hospital, our patients had access to a no-cost assisted reproduction program, and many of the patients with endometriosis and infertility and without associated pelvic pain were referred to this program, which may have contributed to a lower indication of laparoscopy for infertility in this group.

Endometriosis can have a profound impact on women’s lives, including associated pain, infertility, decreased quality of life, and interference with daily life, relationships, and livelihood^18^. The gold standard surgical approach is based on a minimally invasive surgical procedure (laparoscopy)^19^. The laparoscopic approach presents some advantages over open surgery, including reduced trauma, stress, postoperative adhesions, hernia, and hospital stay and shorter recovery time. However, the operative time was longer in the public hospital, which is mainly explained by the fact that these surgeries are performed by resident doctors who, even under the supervision of the preceptors, are learning to perform laparoscopy. The lengths of hospitalization were also significantly higher in teaching hospitals. One possible explanation to justify a longer stay in the public hospital was the high percentage of patients (more than 30%) where the surgery was converted to an open procedure. In our cohort of private patients, the conversion rate to laparotomy was 2%, which is very similar to other series^20^.

The rates of hospital readmission, reoperation and need for stoma were not significantly different for either hospital. However, the rate of complications was significantly higher in patients at the university hospital. Major complications, which necessitate another surgery, occurred almost 4 times more frequently in public hospitals. It is known that a learning curve exists for laparoscopic surgery endometriosis procedures. The learning curve is usually defined by the operating time, perioperative complications, and surgical outcome^21^. Thus, the benefits expected from laparoscopic surgery in endometriosis may be limited by harms resulting from surgical inexperience. There are no doubts that we should always perform laparoscopic surgery in DIE, with or without bowel involvement. However, it is important to emphasize that in public teaching hospitals, laparoscopic procedures are performed by resident doctors, and the rate of complications and the time of operation may decrease and the potential for benefit may increase with time as the surgeon’s experience increases.

Our study has several limitations. First, this study was limited by its retrospective nature (public hospital), although a prospective database was used (in private hospital). In addition, some clinical data were not available in medical records in either hospital, such as opening of the vagina or high anastomosis, anal verge, postoperative fertility rate and recurrence. Second, one bias that may have occurred in our study is the comparison of different groups. There is a large disproportion in number of patients between the two groups. It would be more appropriate to have an equivalent number of cases in both groups. To reduce bias from confounding variables, some authors recommend calculating a propensity score with an inverse probability of treatment weighting to compare the two treatment groups. However, the small number of patients operated on the public hospital during the study period did not allow for statistical analysis by propensity score matching in our study. Finally, long-term postoperative functional outcomes and urinary and/or gastrointestinal disorders (such as abdominal pain, urinary incontinence, fecal incontinence or constipation) were not evaluated in the study. We also did not evaluate the improvement of endometriosis-related symptoms after surgery.

## Conclusion

Laparoscopic surgery in private centers was associated with fewer major complications and reduced surgical times, lengths of stay and rates of conversion to open surgery when compared to that in public teaching hospitals.
